# On dogs, people, and a rabies epidemic: results from a sociocultural study in Bali, Indonesia

**DOI:** 10.1186/s40249-015-0061-1

**Published:** 2015-06-30

**Authors:** Maria Digna Winda Widyastuti, Kevin Louis Bardosh, ᅟ Sunandar, C. Basri, E. Basuno, A. Jatikusumah, R. A. Arief, A. A. G. Putra, A. Rukmantara, A. T. S. Estoepangestie, I. Willyanto, I. K. G. Natakesuma, I. P. Sumantra, D. Grace, F. Unger, J. Gilbert

**Affiliations:** Center for Indonesian Veterinary Analytical Studies, Bogor, Indonesia; School of Social and Political Science, The University of Edinburgh, Edinburgh, UK; Faculty of Veterinary Medicine, Bogor Agricultural University, Bogor, Indonesia; Center for Socio Economic and Agricultural Policy Studies, Ministry of Agriculture, Bogor, Indonesia; Disease Investigation Centre Denpasar, Ministry of Agriculture, Denpasar, Indonesia; Edelman Indonesia, Jakarta, Indonesia; Faculty of Veterinary Medicine, Airlangga University, Surabaya, Indonesia; InI Veterinary Service, Surabaya, Indonesia; Bali Provincial Livestock and Animal Health Services Office, Denpasar, Indonesia; International Livestock Research Institute, Nairobi, Kenya

**Keywords:** Bali, Rabies, Sociocultural, KAPs, Community-driven activities

## Abstract

**Background:**

Previously free of rabies, Bali experienced an outbreak in 2008, which has since caused a large number of human fatalities. In response, both mass dog culling and vaccination have been implemented. In order to assess potential community-driven interventions for optimizing rabies control, we conducted a study exploring the relationship between dogs, rabies, and the Balinese community. The objectives of this study were to: i) understand the human-dog relationship in Bali; ii) explore local knowledge, attitudes, and practices (KAPs) relating to rabies; and iii) assess potential community-driven activities to optimize rabies control and surveillance.

**Methods:**

Conducted between February and June 2011, the study combined a questionnaire (*n* = 300; CI = 95 %; error margin = 5 %) and focus group discussions (FGDs) in 10 villages in the Denpasar, Gianyar, and Karangasem regencies. The questionnaire included a Likert scale to assess community knowledge and attitudes. For the knowledge assessment, three points were given for a correct answer, while wrong answers and uncertain answers were given zero points. For the attitudes assessment, three points were given for a positive answer, two points for a neutral answer, and one point for a negative answer. Respondent knowledge was categorized as good (score >40), fair (score 20–40), or poor (score <20), based on a maximum total score 60. Respondent attitudes were categorized as positive (score >26), neutral (score 13–26), or negative (score <13), based on a maximum total score of 39. Mixed-gender FGDs in each sub-village (*banjar*) were conducted, each involving 7–15 participants to complement the questionnaire results. On a follow-up research trip in mid-2013, the data analysis was triangulated and validated using semi-structured interviews. Questionnaire data were analyzed descriptively using SPSS 17.0, while qualitative data from interviews and FGDs were analyzed manually according to accepted methods of coding and memo writing. The chi-square test was then used to analyze the statistical relationships between knowledge and attitudes of the respondents.

**Results:**

Out of the total 300 respondents, most were predominantly male (82 %), Hindu (99 %), married (96 %), older than 30 years of age (92 %), and owned dogs (72 %). Dog ownership was motivated by culture, personal taste, and function, with dogs was being used as guards (85 %) and companion animals (27 %), and was sometimes related to religious or traditional obligations (2 %). Relating to their culture and local beliefs, and eventually becoming their way of life, 79 % of respondents kept free-roaming dogs. With the rabies outbreak in Bali and Western breeds becoming more popular, more responsible dog ownership (leashing, confining, regular feeding) became more acceptable and changed community perceptions on keeping dogs, even though the sustainability of this practice cannot be gauged. In addition, the economic situation posed major problems in rural areas. The level of community knowledge about rabies and its associated control programs were generally fair and community attitudes were positive. However, community KAPs still need to be improved. A total of 74 % respondents reported to have vaccinated their dogs in 2011, but only few were found to report rabid animals to livestock officers (12 %) and a significant number believed that washing a bite wound was not important (62 %). Moreover, free-roaming dog practices and discarding of unwanted female puppies still continue and possibly create difficulties for rabies elimination as these practices potentially increase the stray dog population. We identified three major sociocultural aspects with potential for community-driven interventions to optimize current rabies elimination efforts: integrating local notions of *ahimsa* (non-violence) into education campaigns, engaging communities through the local *banjar* sociopolitical system, and working with traditional legal structures to increase local compliance with rabies control.

**Conclusion:**

The human-dog relationship in Bali is multifaceted. Due to the uniqueness of the culture and the local beliefs, and encouraged by a socioeconomic aspect, a number of local practices were found to be constituting risk factors for continued rabies spread. Community knowledge and attitudes, which can consequently result in behavioral changes, needs to be improved across different genders, ages, educational backgrounds, and roles in the community, regardless of the individual village’s experiences with rabies. Furthermore, community-driven activities based on sociocultural conditioning and community capacity at the *banjar* and village levels, such as public awareness activities, vaccination, dog registration, dog population management, and rapid response to dog bites, were identified as being able to complement the rabies control program in Bali. The program also needs recognition or acknowledgement from governments, especially local government as well as regular mentoring to improve and sustain community participation.

**Electronic supplementary material:**

The online version of this article (doi:10.1186/s40249-015-0061-1) contains supplementary material, which is available to authorized users.

## Multilingual abstracts

Please see Additional file 1 for translations of the abstract into the six official working languages of the United Nations.

## Background

Rabies is a significant but neglected disease predominantly transmitted through dog bites and responsible for an estimated 55,000 to more than 70,000 human deaths annually worldwide, but occurring mostly in Asia and Africa [1–4]. The rabies virus is endemic in 24 of the 33 Indonesian provinces where there have been numerous outbreaks in the last few decades [5–7]. A popular tourist destination, the island of Bali, remained free from rabies until 2008, but in November of that year, fishermen travelling with an infected dog from a neighboring island introduced the virus. The initial emergency response attempted to contain the disease to the southern peninsula by vaccinating dogs (the most accepted public health strategy for rabies control) and eliminating unconfined or free-roaming dogs, but the disease nevertheless quickly spread throughout the island. A much larger campaign then followed which, apart from canine vaccination, also included the mass culling of 100,000 dogs. This provoked opposition from international rabies experts and animal welfare groups who argued that culling is not only inhumane but also ineffective for controlling the rabies virus [3, 8]. Additionally, the lengthy campaign time lowered population immunity through high population turnover and the short life of the vaccine that was used [9, 10]. In late 2010, a local non-governmental organization (NGO), the Bali Animal Welfare Association (BAWA), together with the Balinese government, initiated an island-wide canine vaccination campaign, vaccinating 250,000 dogs over six months by using an innovative technique to deal with the massive free-roaming dog population: dog-catcher teams. This achieved an estimated coverage of more than 70 %, which is the accepted coverage target to sustainably reduce virus circulation. Two additional mass vaccination rounds were then conducted by the Balinese government and the Food and Agriculture Organization of the United Nations (FAO) in 2011 and 2013, with a corresponding reduction in human rabies fatalities and reported rabid dogs [5]. This encouraged the Balinese local government to set 2015 as the official target year for rabies elimination.

Since 2008, rabies has cost the Balinese government more than US $13 million, led to the death of more than 150 people, and has been responsible for tens of thousands of expensive human post-exposure prophylactic (PEP) treatments that are given after suspected rabid dog bites [5]. Bali is a densely populated island inhabited by some four million people and before the introduction of rabies had one of the highest dog densities in the world; the estimated dog population was between 400,000 and 800,000 before the rabies outbreak [9–12].

Ubiquitous around markets, temples, beaches, garbage dumps, and rice paddies, the majority of Bali’s dogs are owned, free-roaming, indigenous street dogs. Known to be independent, aggressive, and territorial, the Balinese street dog remains one of the most genetically diverse canine populations, related to the Australian Dingo and Chow Chow [13, 14]. A small proportion of the overall dog population, the Kintamani dog of Bali (an emerging breed unique to the Kintamani region), contrasts significantly with the wider canine population, in that these dogs show physical affection to people, and climb roofs, garden walls, and trees [14]. With the increase in tourism in the mid-1990s [15], Western dog breeds have also become increasingly popular but are largely confined to urban areas. For many of Bali’s free-roaming dogs, these socioeconomic changes together with a high birth rate have led to many dogs being neglected, malnourished, and suffering from skin diseases [12, 16].

Aside from a unique dog population, Bali has also long been known for its unique culture, arts, and religion, and a diverse amount of anthropological research has been conducted on the island since the early Dutch colonial era. Much of this work has shown the interrelatedness between Balinese religion, society, and the natural world; for instance, the importance of water temples in regulating irrigation networks used in rice cultivation as well as the central role of the Balinese cockfight to notions of identity and socialization [17, 18]. A mixture of traditional Hinduism and Buddhist and animist elements, Balinese Hinduism is both deeply communal and ritually prescriptive, orientated around the need to maintain harmony between humans, nature, and the spiritual realm through daily sacrifices to both gods and demons, some of which involve animals [19]. Religious cosmology and iconography make significant use of animal motifs in depicting the world of spirits, including the abundant use of teeth and fangs [20].

Due to the island’s dense dog population, current efforts to eliminate rabies from Bali have received much international attention. If successful, the Balinese elimination campaign could help set a precedent for the feasibility of rabies elimination through mass dog vaccination in Asia [10]. While a number of studies have examined sociocultural aspects to dog ownership and rabies control in the region (including in Bhutan, Cambodia, India, Sri Lanka, and Vietnam) [21–25], there is a dearth of such information available from the unique Balinese context. There have also been surprisingly few knowledge, attitudes, and practices (KAPs) studies that have used qualitative methods or explored potential community-driven strategies in relation to rabies more generally [26]. The aims of this study, therefore, were to: i) understand the human-dog relationship in Bali; ii) explore local KAPs relating to rabies; and iii) assess potential community-driven activities to optimize rabies control and surveillance.

## Methods

**Fig. 1:**
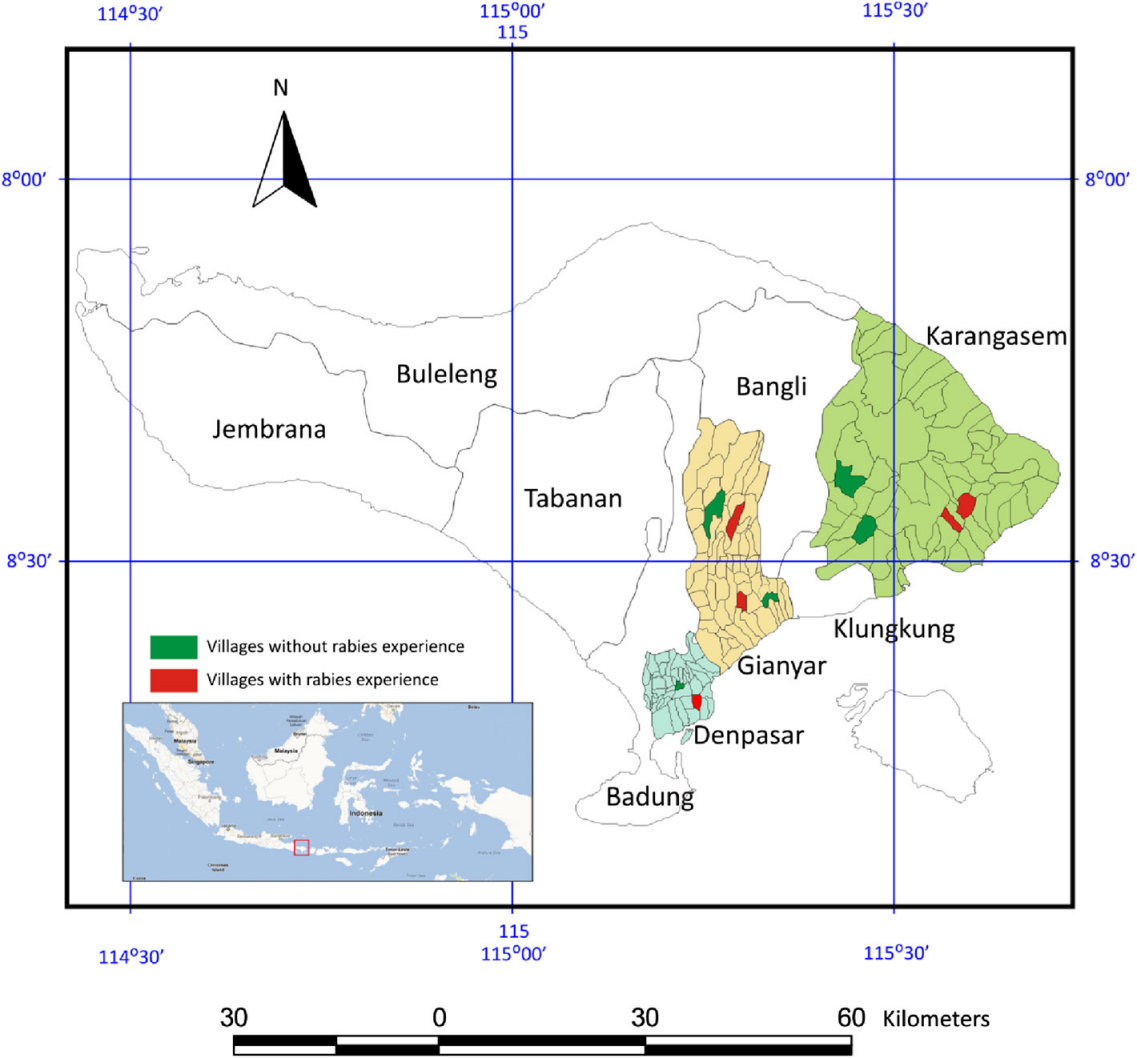
Study location, Bali, Indonesia

This cross-sectional study was conducted from February to June 2011 in 10 *banjars* (sub-villages) selected purposively from 10 villages in Denpasar city, and the Gianyar and Karangasem districts. These areas were chosen due to their demographic and economic differences, representing an urban, suburban, and rural district, respectively (see Fig. 1 and Table 1). Based on government records, half of the selected *banjars* had experienced both human and canine rabies, while half remained free from rabies. A questionnaire exploring knowledge, attitudes, and practices (KAPs) towards dogs, rabies, and canine vaccination was first administered, which used both open and closed-ended questions. A total of 30 participants per *banjar* (*n* = 300; CI = 95 %; error margin = 5 %) participated in the questionnaire, with a proportional number of government officers, religious leaders, and members of the general population being selected in each village.

**Table 1:** Study location in the Bali province, by villages with and without rabies experience

Villages status	No.	Villages	District/city
With rabies experience	1	Renon	Denpasar
	2	Blahbatuh	Gianyar
	3	Kenderan	Gianyar
	4	Bungaya Kauh	Karangasem
	5	Padang Kerta	Karangasem
Without rabies experience	1	Dangin Puri	Denpasar
	2	Abianbase	Gianyar
	3	Kelusa	Gianyar
	4	Menanga	Karangasem
	5	Sindhuwati	Karangasem

The questionnaire included a Likert scale to assess knowledge and attitudes using a scoring system. For the knowledge assessment, three points were given for a correct answer, while wrong answers and uncertain answers were given zero points. Respondent knowledge was categorized as good (score >40), fair (score 20–40), or poor (score <20), based on a maximum total score of 60. For the attitude assessment, three points were given for a positive answer, two points for a neutral answer, and one point for a negative answer. Respondent attitudes were categorized as positive (score >26), neutral (score 13–26), or negative (score <13), based on a maximum total score of 39. In order to triangulate and further explore, we subsequently conducted interviews as well as one mixed-gender focus group discussion (FGD) in each *banjar*, which lasted between one to two hours and involved 7–15 participants. The participants were often the same people who had answered our previous questionnaires, but the FDGs also included other key persons, such as traditional leaders and village leaders. The FGDs revisited the same questions as our questionnaires but allowed for much more nuanced explorations of emerging themes and sub-themes. During a follow-up research trip in mid-2013, semi-structured interviews were used to triangulate and validate the data analysis.

Questionnaire data were analyzed descriptively using SPSS 17.0, while qualitative data from interviews and FGDs were analyzed manually according to accepted methods of coding and memo writing. The chi-square test was then used to analyze the statistical relationships between knowledge and attitudes of the respondents. According to our system, approval for this study was obtained from the Political Unity and Nation Agency at Bali Province and Livestock Services Office of Bali Province, and acknowledged by the Directorate of Animal Health, Directorate General of Livestock and Animal Health, Ministry of Agriculture, Republic of Indonesia.

## Results

### Dog ownership and management practices

Slightly less than three-quarters of the questionnaire respondents (72 %) reported to own a dog. Out of the total number of respondents, most were predominantly male (82 %), Hindu (99 %), married (96 %), and older than 30 years of age (92 %). The respondents’ demographic profiles are presented in Table 2. It was not intentional to choose a larger number of male respondents, but this is what happened when convenience sampling was done for the household level. More than 50 % of the respondents (59 %) either graduated from high school or attended university, and 65 % had an important role in the community, such as being in government or in a traditional leader role. Roughly half of the respondents considered dog ownership integral to both Balinese and Hindu traditions, though not necessarily part of religious obligation. Table 3 shows dog ownership patterns in villages with and without rabies cases, revealing no clear distinctions between these groups. In focus groups and interviews, dog ownership was discussed in more detail, and was linked to culture, personal taste, and local livelihoods.

**Table 2:** Respondents’ demographic profiles

No.	Variables	Villages with rabies experience (*n* = 150)		Villages without rabies experience (*n* = 150)		Total (*n* = 300)	
		*n*	%	*n*	%	*n*	%
1	Sex						
	▪ Male	118	79	129	86	247	82
	▪ Female	32	21	21	14	53	18
	Total	150	100	150	100	300	100
2	Age						
	▪ <30 years old	18	12	7	5	25	8
	▪ 30–50 years old	83	55	97	64	180	60
	▪ >50 years old	49	33	46	31	95	32
	Total	150	100	150	100	300	100
3	Religion						
	▪ Hindu	148	99	149	99	297	99
	▪ Moslem	2	1	1	1	3	1
	Total	150	100	150	100	300	100
4	Marital status						
	▪ Married	140	93	147	98	287	96
	▪ Single	10	7	3	2	13	4
	Total	150	100	150	100	300	100
5	Educational background						
	▪ No formal education	9	6	5	3	14	4
	▪ Elementary school (graduated)	40	27	34	23	74	25
	▪ Junior high school (graduated)	20	13	15	10	35	12
	▪ High school (graduated)	44	30	64	43	108	36
	▪ University	37	24	32	21	69	23
	Total	150	100	150	100	300	100
6	Occupation						
	▪ Farmer (agricultural)	27	18	32	22	59	19
	▪ Housewife	12	8	21	14	33	11
	▪ Government officer	89	60	78	52	167	56
	▪ Works at a private company	2	1	2	1	4	1
	▪ Entrepreneur	5	3	6	4	11	4
	▪ Unemployed	15	10	11	7	26	9
	Total	150	100	150	100	300	100
7	Role in local community						
	▪ Local government	46	31	50	33	96	32
	▪ Traditional leader	49	32	50	33	99	33
	▪ Part of general community	55	37	50	33	105	35
	Total	150	100	150	100	300	100

**Table 3:** Dog ownership patterns, by villages with and without rabies experience

Characteristic	Villages with rabies experience (*n* = 150)		Villages without rabies experience (*n* = 150)	
	*n*	%	*n*	%
Have a dog as it’s part of the Balinese tradition	84	56	89	59
Have a dog as it’s part of the Hindu tradition	75	50	77	51
Keep free-roaming dog(s)	122	81	116	77
Keep a dog to guard the house	122	81	132	88
Dogs sleep around your property	150	100	149	99
You provide food for your dog	147	98	147	98
Type of food provided:				
▪ Commercial food	5	3	9	6
▪ Leftover food	26	17	33	22
▪ A mixture	120	80	107	71
You can handle your dog	144	96	146	97
Preference for dog gender				
▪ Male	116	77	113	75
▪ Female	5	3	12	8
▪ Either	32	21	26	17
Feeling discomfort/fear of stray dogs	134	89	134	89

According to focus group participants, dogs were a ubiquitous part of the Balinese landscape and people were accustomed to having many free-roaming dogs around their fields, streets, homes, and markets since childhood. Representative of the influence of Hinduism, the popular story of King Yudhistira in the Hindu epic *The Mahabharata* was continuously invoked to explain why the Balinese respected and tolerated so many free-roaming dogs. The story goes that after enduring the death of his family on a perilous journey to the Gates of Heaven, an ill-kept dog faithfully accompanies King Yudhistira and when the dog (considered spiritually unclean) is refused entry into the celestial city by the God Indra, Yudhistira refuses to enter without the dog. At this point, the dog transformed into the God Dharma (representing righteousness and justice) and Yudhistira is commended for his loyalty. In the words of one male focus group participant:

“Keeping dogs for the Balinese is just part of the way we are…we respect dogs due to the story of Yudhistira…they have a number of functions but many times people just want dogs around and they feel that life is quiet, or something is missing or not complete if a dog is not there. We are used to having so many dogs around since we were young”.

Considered *“owned but free-roaming”,* the Balinese dog was described as *“semi-wild”, “fiercely independent”, “untamable”,* and *“very territorial”,* spending most of the day roaming within one to four kilometers, and some only returning to their owner compound at night. While dogs were fed a mixture of leftover and commercial dog food, they also ate from local hotels, restaurants, religious offerings, and garbage dumps. Due to recent health messaging campaigns, the majority of the respondents (84 %) felt that dogs should be caged and put on leashes, although these recommendations have not been widely put into practice. The study showed that 79 % of respondents kept their dogs roaming free. Leashed dogs were perceived to be too aggressive and confining dogs was, in the words of one interviewee, *“against the spirit of the Balinese dog*”. Although most owners reported that they could handle their dogs within the family compound (97 %), focus group participants emphasized that this only lasted a few seconds before the dog resisted. Those who fed the dog were usually the same ones that could touch it—and most physical contact was confined to the time when the dog was a puppy.

Not all owned dogs were indigenous Balinese street dogs. Although mostly found in the southern tourist region (including Denpasar and Gianyar), Western breeds were considered to be confined on leashes and kept within the home as companion animals. The population of these companion dogs has increased since the 1990s in parallel to private veterinary practices in Bali and the growth of the expat community. A second significant characteristic of the dog population is the presence of stray dogs, thought to range from 2 to 10 % in the study villages. Considered more aggressive and prone to physically harming people, stray dogs caused discomfort and fear among community members and were widely believed to be responsible for the spread of rabies in Bali. People emphasized that the stray population was maintained by the growth of open garbage dumps (having grown in parallel with the tourism industry), as well as the local practice of discarding unwanted female puppies. In total, 76 % of the questionnaire respondents who had dogs preferred male dogs due to their perceived superior guard dog abilities and the fact that in mating season, packs of unruly males gather at the homes of female dogs, causing a great nuisance to their owners. While male dogs could be given to friends as gifts, female puppies were generally not appreciated. However, unwanted females were not killed outright but often discarded in garbage areas, near restaurants and waterways. This practice was considered widespread throughout the island and motivated by the Hindu principle of *ahimsa* (non-violence) towards the unwanted puppies. However, this local practice was also thought to maintain the stray dog population.

### The role of dogs in Balinese society

Questionnaire respondents reported to use dogs as guards (85 %) and some as companion animals (27 %), while only 2 % related dog ownership to religious or traditional obligations. The idea of dogs being used for recreational purposes was seen as a recent phenomenon. As foreign breeds have been increasingly imported, it is not uncommon to see both expats and the Balinese with small non-indigenous breeds on their scooters in urban areas; what one interviewee called *“the Paris Hilton poodle”,* alluding to a popular Western celebrity. Western breeds were linked to symbols of affluence and modernity while they were also generally considered much better companion animals. Despite this, the Balinese dog was believed to be superior in a number of areas. The most widely cited was that they barked louder and more often at strangers, making them better guard dogs. During focus groups, it became clear that dogs function more as an *“alarm bell”* alerting family members to strangers than as attack dogs, as crime was believed to be low and the dogs were absent (roaming) for most of the day. Farmers living near mountainous areas also reported the use dogs for hunting small animals and for protecting agricultural land from monkeys.

There was also a second, more nuanced way that dogs played the role of an *alarm bell.* Dogs were considered to be able to “*see”,* “*sense”,* and “*know”* when evil spirits were around the home and acted as *de facto “evil spirit alarms”.* Alerted by unusual howling, people would reportedly pray for protection, stay indoors, or avoid some future activity to evade calamity. These types of events were believed to coincide with specific times in the Balinese calendar when the movement of spirits was believed to be more prevalent. As one interviewee, a male village leader, noted:

“A dog is a type of alarm bell for spirits because it is more sensitive than people to the negative powers and can see sorcerers approaching you. Some people believe it is because dogs have evil spirits inside of them, but I disagree. Dogs are loyal creatures and want to warn their owners of the dangers…you find that on certain times of the month, on sacred days such as *Kajeng Kliwon* when spirits roam, you find the dogs are howling like mad”.

It was found that some groups of people also eat dogs, a practice that is widespread in other areas of Indonesia including North Sumatra, North Sulawesi, and East Nusa Tenggara. During the initial rabies outbreak, there were reports that dogs were being stolen and moved to northern Bali to be served in restaurants, which may have facilitated the spread of the virus. While it was difficult to confirm this, focus groups and interviews gave varying reports about the popularity of dog meat. Some people commented that it was widely consumed until the rabies epidemic created a fear of transmission, while others reported that it was only practiced in areas with migrant non-Balinese populations. There were few reasons given for the consumption of dog meat. The most accepted was that certain parts of a dog were eaten under the supervision of a traditional doctor for medical conditions, including a dog’s heart for the treatment of asthma. The second, albeit a less discussed idea, related to the consumption of dog for the increase of the male libido, linked to the belief that dog meat could confer magical powers and increase sexual dexterity. To some, however, dogs were synonymous with evil spirits and these respondents were opposed to eating dogs on religious grounds, arguing that this practice should be avoided because, in the words of one focus group participant,*“…it would make you dirty and destroy your aura since evil spirits can be inside dogs and this should be avoided”.*

Dogs also played a very specific role in religious ceremonies. Contrary to Indian Hinduism, the Balinese use animals in their religious sacrifices, including dogs. The *Bhuta Yadnya* is the practice of providing specific types of ritual sacrifices of flowers and food to feed and show respect to evil spirits that are believed to hide in natural objects and places. Such sacrifices appease these demons, at which point they turn into guards that defend the household from other spirits. The sacrifice that involves dogs is believed to target evil spirits residing throughout the *banjar* area and is done when advised under the direction of a priest. This was reported to occur numerous times every year in each of the study villages by different households. The type of dog needed was the *blang bungkem,* a dog with reddish brown fur and a black snout, chosen because its red color corresponds to the symbolic color of the evil spirit targeted for such sacrifices. There was no reported breeding of the *blang bungkem*; this breed was considered relatively rare to find but could be bought at certain markets if not found within the community. An elaborate ceremony, which can cost many hundreds of dollars to conduct, is involved in the sacrifice where the body parts of the dog are offered up to the evil spirit(s). Interviewed priests explained that the dog then attains a higher state of re-incarnation as spiritual balance is returned to the community.

### Community knowledge and attitudes towards rabies

Considered a dangerous and fatal disease, 97 % of the questionnaire respondents had heard of rabies (see Table 4). Respondents knew that rabies could be transmitted by dogs (95 %), cats (61 %), monkeys (44 %), and bats (6 %), but a few wrongly believed that rabies can be transmitted by chickens (0.6 %), mosquitoes (1 %), and flies (0.6 %). Transmission was thought to be largely through bites (96 %), which was believed to be the common way of disease transmission. Only a few respondents understood that licks (18 %) and scratches (15 %) also had the potential to transmit the disease. Focus groups revealed that people had varying degrees of familiarity with canine rabies. The most commonly described symptoms included: aggression, hyperactivity, salivation, and fear of water and light. Major sources of information on rabies included television and radio (68 %), neighbors (27 %), print media (24 %), and livestock officers (20 %).

**Table 4:** Knowledge and experience of rabies, by villages with and without rabies experience

Characteristic	Villages with rabies experience (*n* = 150)		Villages without rabies experience (*n* = 150)	
	*n*	%	*n*	%
Had heard of rabies	146	97	146	97
Which animals can transmit rabies:				
▪ Dogs	143	95	143	95
▪ Cats	83	55	101	67
▪ Monkeys	54	36	77	51
▪ Bats	5	3	14	9
How rabies can be transmitted:				
▪ Bite	141	94	146	97
▪ Lick	29	19	26	17
▪ Scratch	26	17	20	13
Source of information to learn about rabies:				
▪ Broadcast media (TV & radio)	98	65	105	70
▪ Neighbors	48	32	33	22
▪ Print media	29	19	42	28
▪ *Banjar* meetings	24	16	26	17
▪ Livestock officers	24	16	36	24
▪ NGOs	5	3	12	8
▪ Internet	0	0	2	1
Seeking medication if bitten by suspected dogs	131	87	128	85
Washing the wound if bitten by suspected rabid dog	53	35	62	41
Reporting to livestock services if bitten by suspected rabid dog	21	14	14	9
Action if suspected rabid dog is identified:				
▪ Catch it	21	14	17	11
▪ Kill it	113	75	116	77
▪ Let it go	17	11	18	12
Believe that canine vaccination can eliminate rabies from Bali	147	98	137	91
Believe that culling stray dogs can eliminate rabies from Bali	128	85	120	80
Had their dogs vaccinated for rabies	110	73	111	74
If PEP is provided in your community:				
▪ It’s free	71	47	63	42
▪ There is a cost	30	20	36	24
▪ Don’t know	50	33	51	34
Dog culling had taken place in your village	122	81	99	66

Most respondents (86 %) believed that visiting a local hospital or clinic was necessary after a suspected rabid dog bite and that injection and/or vaccinations (post-exposure prophylactic [PEP]) should be sought. More than half of the respondents (61 %) wrongly believed there was a medicine to treat rabies. Only 39 % understood that injections and/or vaccinations can only block the virus transmission, and was not the same as medicine. While PEP treatment was freely available in the outbreak situation, 22 % of the questionnaire respondents believed there was a fee involved, while 34 % did not know whether there was a fee or not.

A significant number of respondents (62 %) did not think that washing a wound from a suspected rabid dog bite was important. Our results show that respondents understood that they should visit a hospital or get treatment, but didn’t really consider this to be a matter that required urgent treatment. Moreover, a widespread local belief (held prior to the rabies outbreak) was that dog licks could heal wounds and this led to the practice of not washing wounds, including bite wounds. Only 12 % of the respondents reported that it was important to consult with local livestock officers in case of dog bites. This small number could be attributed to the fact, at least partially, that most people (76 %) reported that killing a suspected rabid dog was the immediate community response, with only a small number believing that rabid dogs should be caught (13 %) or let go (12 %). A total of 17 % reported that they had previously been bitten by a dog.

Based on the scoring system, more than a half of the total respondents (*n* = 300, 62 %) had a fair knowledge about rabies (see Table 5). Similar results were found both in the villages with (66 %) and without (57 %) rabies experience (see Table 6). From the total number of respondents, 36 % had a good knowledge, while only 2 % had a poor knowledge about rabies. Based on the village status, less than 5 % of the respondents had a poor knowledge about rabies (3 % in the villages with rabies experience; 2 % in the villages without rabies experience), and more than a quarter of respondents had a good knowledge about rabies (31 % in the villages with rabies experience; 41 % in the villages without rabies experience).

**Table 5:** Level of community knowledge about rabies and the rabies control program (*n* = 300)

No.	Variables	Level of knowledge								*p*-value
		Good		Fair		Poor		Total (*n* = 300)		
		*n*	%	*n*	%	*n*	%	*n*	%	
1	Sex									0.156
	▪ Male	92	37	151	61	4	2	247	100	
	▪ Female	16	30	34	64	3	6	53	100	
	Total	108	36	185	62	7	2	300	100	
2	Age									0.187
	▪ <30 years old	10	40	14	56	1	4	25	100	
	▪ 30–50 years old	73	41	103	57	2	2	180	100	
	▪ >50 years old	25	26	68	72	4	2	95	100	
	Total	108	36	185	62	7	2	300	100	
3	Religion									0.390
	▪ Hindu	108	36	182	61	7	3	297	100	
	▪ Moslem	0	0	3	100	0	0	3	100	
	Total	108	36	185	62	7	2	300	100	
4	Educational background									0.002^1^
	▪ No formal education	3	21	10	72	1	7	14	100	
	▪ Elementary school (graduated)	15	20	54	73	5	7	74	100	
	▪ Junior High school (graduated)	12	34	23	66	0	0	35	100	
	▪ High school (graduated)	45	42	63	58	0	0	108	100	
	▪ University	33	48	35	51	1	1	69	100	
	Total	108	36	185	62	7	2	300	100	
5	Role in local community									0.034^1^
	▪ Local government	46	48	49	51	1	1	96	100	
	▪ Traditional leader	28	28	69	70	2	2	99	100	
	▪ Part of general community	34	32	67	64	4	4	105	100	
	Total	108	36	185	62	7	2	300	100	

^1^: statistically significant at 95 % confidence level

**Table 6:** Level of community knowledge towards rabies and the rabies control program, based on village status (*n* = 150)

No.	Variables based on village status			Level of knowledge								*p*-value
				Good		Fair		Poor		Total (*n*=150)		
				*n*	%	*n*	%	*n*	%	*n*	%	
1	Sex	Village with rabies experience	▪ Men	39	33	76	64	3	3	118	100	0.682
			▪ Women	8	25	23	72	1	3	32	100	
			Total	47	31	99	66	4	3	150	100	
		Village without rabies experience	▪ Men	53	41	75	58	1	1	129	100	0.029^1^
			▪ Women	8	38	11	52	2	10	21	100	
			Total	61	41	86	57	3	2	150	100	
2	Age	Village with rabies experience	▪ <30 years old	7	39	11	61	0	0	18	100	0.494
			▪ 30–50 years old	29	35	52	63	2	2	83	100	
			▪ >50 years old	11	22	36	74	2	4	49	100	
			Total	47	31	99	66	4	3	150	100	
		Village without rabies experience	▪ <30 years old	3	43	3	43	1	14	7	100	0.045^1^
			▪ 30–50 years old	44	45	51	53	2	2	97	100	
			▪ >50 years old	14	30	32	70	0	0	46	100	
			Total	61	41	86	57	3	2	150	100	
3	Religion	Village with rabies experience	▪ Hindu	47	32	97	65	4	3	148	100	0.593
			▪ Moslem	0	0	2	100	0	0	2	100	
			Total	47	31	99	66	4	3	150	100	
		Village without rabies experience	▪ Hindu	61	41	85	57	3	2	149	100	0.688
			▪ Moslem	0	0	1	100	0	0	1	100	
			Total	61	41	86	57	3	2	150	100	
4	Educational background	Village with rabies experience	▪ No formal education	2	22	6	67	1	11	9	100	0.053
			▪ Elementary school (graduated)	6	15	31	77	3	8	40	100	
			▪ Junior high school (graduated)	7	35	13	65	0	0	20	100	
			▪ High school (graduated)	18	41	26	59	0	0	44	100	
			▪ University	14	38	23	62	0	0	37	100	
			Total	47	31	99	66	4	3	150	100	
		Village without rabies experience	▪ No formal education	1	20	4	80	0	0	5	100	0.107
			▪ Elementary school (graduated)	9	26	23	68	2	6	34	100	
			▪ Junior high school (graduated)	5	33	10	67	0	0	15	100	
			▪ High school (graduated)	27	42	37	58	0	0	64	100	
			▪ University	19	59	12	38	1	3	32	100	
			Total	61	41	86	57	3	2	150	100	
5	Role in local community	Village with rabies experience	▪ Local government	20	43	26	57	0	0	46	100	0.176
			▪ Traditional leader	11	22	36	74	2	4	49	100	
			▪ Part of general community	16	29	37	67	2	4	55	100	
			Total	47	31	99	66	4	3	150	100	
		Village without rabies experience	▪ Local government	26	52	23	46	1	2	50	100	0.183
			▪ Traditional leader	17	34	33	66	0	0	50	100	
			▪ Part of genera; community	18	36	30	60	2	4	50	100	
			Total	61	41	86	57	3	2	150	100	

^1^: statistically significant at 95 % confidence level

A range of educational statuses were represented among the respondents: 4 % never attended school, 25 % completed elementary school, 12 % completed junior high school, 36 % completed senior high school, and 23 % completed university (see Table 2). It was observed that there was a significant relationship between knowledge and educational background (*p*-value = 0.002) (see Table 5). Table 5 also shows the significant relationship that was observed between knowledge and the role of respondents in the community (*p*-value = 0.034), even though there was no significant variation in the range of the respondents’ roles: 32 % worked for the local government, 33 % were traditional leaders, and 35 % were just part of the general community (see Table 2). Moreover, based on village status, there was no significant relationship observed between knowledge and both of these variables (see Table 6).

Based on the village status, there was a significant relationship observed between knowledge and the variables of sex and age (sex, *p*-value = 0.029 and age, *p*-value = 0.045) in villages without rabies experience, while no significant relationship was found between knowledge and these variables in villages with rabies experience.

The majority of the respondents had positive attitudes towards the rabies control program (96 %), while 4 % had neutral feelings towards it, and no one showed a negative attitude (see Table 7). Similar results were found in the different villages, but the majority of respondents had positive attitudes (96 % in villages with rabies experience, 95 % in villages without rabies experience), and only a small proportion was neutral (4 % in villages with rabies experience, 5 % in villages without rabies experience) (see Table 8). No significant relationship was observed (*p*-value > 0.05) between respondents’ attitudes and the examined variables (sex, age, religion, educational background, role in local community), both based on the total number of respondents and the village status.

**Table 7:** Level of community attitudes towards rabies and the rabies control program (*n* = 300)

No.	Variables	Level of attitude						*p*-value
		Positive		Neutral		Total (*n* = 300)		
		*n*	%	*n*	%	*n*	%	
1	Sex							0.371
	▪ Male	238	96	9	4	247	100	
	▪ Female	49	92	4	8	53	100	
	Total	287	96	13	4	300	100	
2	Age							0.513
	▪ <30 years old	25	100	0	0	25	100	
	▪ 30–50 years old	172	96	8	4	180	100	
	▪ >50 years old	90	95	5	5	95	100	
	Total	287	96	13	4	300	100	
3	Religion							1.00
	▪ Hindu	284	96	13	4	297	100	
	▪ Moslem	3	100	0	0	3	100	
	Total	287	96	13	4	300	100	
4	Educational background							0.343
	▪ No formal education	14	100	0	0	14	100	
	▪ Elementary school (graduated)	72	97	2	3	74	100	
	▪ Junior high school (graduated)	35	100	0	0	35	100	
	▪ High school (graduated)	102	94	6	6	108	100	
	▪ University	64	93	5	7	69	100	
	Total	287	96	13	4	300	100	
5	Role in local community							0.170
	▪ Local government	89	93	7	7	96	100	
	▪ Traditional leader	95	96	4	4	99	100	
	▪ Part of general community	103	98	2	2	105	100	
	Total	287	96	13	4	300	100

**Table 8:** Level of community attitude towards rabies and the rabies control program based on village status (*n* = 150)

No.	Variables based on village status			Level of attitude						*p*-value
				Positive		Neutral		Total (*n*=150)		
				*n*	%	*n*	%	*n*	%	
1	Sex	Village with rabies experience	▪ Men	114	97	4	3	118	100	0.823
			▪ Women	30	94	2	6	32	100	
			Total	144	96	6	4	150	100	
		Village without rabies experience	▪ Men	124	96	5	4	129	100	0.562
			▪ Women	19	90	2	10	21	100	
			Total	143	95	7	5	150	100	
2	Age	Village with rabies experience	▪ <30 years old	18	100	0	0	18	100	0.639
			▪ 30–50 years old	79	95	4	5	83	100	
			▪ >50 years old	47	96	2	4	49	100	
			Total	144	96	6	4	150	100	
		Village without rabies experience	▪ <30 years old	7	100	0	0	7	100	0.683
			▪ 30–50 years old	93	96	4	4	97	100	
			▪ >50 years old	43	93	3	7	46	100	
			Total	143	95	7	5	150	100	
3	Religion	Village with rabies experience	▪ Hindu	142	96	6	4	148	100	1.00
			▪ Moslem	2	100	0	0	2	100	
			Total	144	96	6	4	150	100	
		Village without rabies experience	▪ Hindu	142	95	7	5	149	100	1.00
			▪ Moslem	1	100	0	0	1	100	
			Total	143	95	7	5	150	100	
4	Educational background	Village with rabies experience	▪ No formal education	9	100	0	0	9	100	0.820
			▪ Elementary school (graduated)	38	95	2	5	40	100	
			▪ Junior high school (graduated)	20	100	0	0	20	100	
			▪ High school (graduated)	42	96	2	4	44	100	
			▪ University	35	95	2	5	37	100	
			Total	144	96	6	4	150	100	
		Village without rabies experience	▪ No formal education	5	100	0	0	5	100	0.331
			▪ Elementary school (graduated)	34	100	0	0	34	100	
			▪ Junior high school (graduated)	15	100	0	0	15	100	
			▪ High school (graduated)	60	94	4	6	64	100	
			▪ University	29	91	3	9	32	100	
			Total	143	95	7	5	150	100	
5	Role in local community	Village with rabies experience	▪ Local government	43	93	3	7	46	100	0.486
			▪ Traditional leader	47	96	2	4	49	100	
			▪ Part of general community	54	98	1	2	55	100	
			Total	144	96	6	4	150	100	
		Village without rabies experience	▪ Local government	46	92	4	8	50	100	0.350
			▪ Traditional leader	48	96	2	4	50	100	
			▪ Part of general community	49	98	1	2	50	100	
			Total	143	95	7	5	150	100	

We also found no significant relationship between respondents’ knowledge and attitude towards rabies and the rabies control program (*n* = 300) in villages without rabies experience. However, a significant relationship between these variables was found in villages with rabies experience (*p*-value = 0.009) (see Table 9).

**Table 9:** Level of community knowledge and attitudes towards rabies and the rabies control program, based on the respondent group

No.	Respondent/Village group (number of respondents)	Level of knowledge	Level of attitude						*p*-value
			Positive		Neutral		Total		
			*n*	%	*n*	%	*n*	%	
1	All respondents (*n* = 300)	▪ Good	102	94	6	6	108	100	0.274
		▪ Fair	179	97	6	3	185	100	
		▪ Poor	6	86	1	14	7	100	
		Total	287	96	13	4	300	100	
2	Village with rabies experience (*n* = 150)	▪ Good	43	91	4	9	47	100	0.009^1^
		▪ Fair	98	99	1	1	99	100	
		▪ Poor	3	75	1	25	4	100	
		Total	144	96	6	4	150	100	
3	Village without rabies experience (*n* = 150)	▪ Good	59	97	2	3	61	100	0.717
		▪ Fair	81	94	5	6	86	100	
		▪ Poor	3	100	0	0	3	100	
		Total	143	95	7	5	150	100	

^1^: statistically significant at 95 % confidence level

### Attitudes towards vaccination

Most of the respondents (91 %) reported that there was a free vaccination program in their area, and that it had been conducted by the government (66 %) or an NGO (34 %). A high percentage (94 %) agreed that annual vaccination was necessary for rabies control. A total of 74 % claimed to have had their dog(s) vaccinated during the first island-wide vaccination campaign in 2011. Focus group participants were unanimous that the majority of dogs were not vaccinated at central points but rather by dog-catching teams moving around the *banjar*. This was considered a necessary strategy in Bali due to the particular character of the Balinese dog. In the words of one female interviewee:

“You cannot bring these roaming dogs to get vaccinated as they will fight you, and once a dog sees or hears another dog being vaccinated, it will run away because it wants to be free and howl about the pain involved and it will hide…you cannot bring these dogs so dog-catchers must come”.

Similar to a recent study done in Tanzania [26], other reasons stated for the lack of compliance with a central vaccination point involved: people being too busy, owners being unable to handle their dogs, most dogs remaining outside their homes for most of the day, and information about vaccination being disseminated only the night before the campaign. While vaccination coverage was felt to be very high (over 70 %), doubts about the coverage in puppies and stray dogs were widespread.

Community members referred to two main challenges related to dog-catchers. A general perception was that it was becoming exceedingly more difficult to catch dogs during the second (in late 2011) and third (in 2013) vaccination rounds since the dogs were believed to have become *“resistant”* to the catching system, which was ascribed to their traumatic experience of being caught and vaccinated, as well as their *“intelligence”* and *“memory”* of the event. Second, specific ecological areas such as garbage dumps and large forest or agricultural areas were thought to be difficult for dog-catchers, especially when targeting stray dogs. In one of the study villages which had experienced two rabies deaths and many more suspected rabid dogs, over 60 % of the area was covered with snakeskin fruit trees. Snakeskin fruit trees are short, dense, thorny trees obstructing visibility and mobility. Besides guarding houses, dogs are also used to guard these plantations and it was believed that these trees allowed dogs to *“hide”* during the vaccination campaigns due to the remoteness of and inaccessibility to the area.

Interestingly, slightly more than half of the questionnaire respondents (57 %) agreed that rabies vaccination was not too expensive for the community and focus groups consistently highlighted that a significant number of people would be willing to pay for the vaccination of puppies, if this service was not widely available (which it was not).

### Attitudes towards dog culling and sterilization

Most respondents reported that dog culling had taken place in their village (74 %) and that it had been carried out by the government; only 2 % claimed that culling had been done by the community itself. This aversion to culling was explained in reference to the Hindu principle of *ahimsa* (or non-violence) towards all sentient beings. At the first outbreak, government culling targeted any dog that was not tied or left indoors. Many expressed anger at having their animals killed (some of which had been vaccinated) and complained about the large number of corpses left by the culling teams. The culling had generated some reluctance during the first round of vaccinations as people hid their dogs in fear that the vaccination teams would kill them. However, most had accepted and even welcomed the culling, regardless of their religious beliefs, due to the widespread fear of rabies. A total of 83 % of the questionnaire respondents reported that the elimination of stray dogs was necessary to eliminate rabies, despite the acknowledgement that it was difficult to differentiate between an owned and a stray dog, as well as that the stray dog population had not been dramatically affected by the culling. Although culling initially reduced the dog population (which was also perceived to have reduced the number of motorcycle accidents caused by high dog numbers), it was perceived that the stray dog population was again quickly growing. It was found that the traditional methods of canine sterilization were practiced only in isolation (adding bread yeast to dog food, for example). While some expressed reluctance to having their dogs sterilized (due to a perception that it made them less aggressive), there was an expectation that the government should conduct more sensitization for the benefit of population control, provide free sterilization services, and address the problem of stray dogs.

### The impact of rabies on dog ownership

The introduction of rabies in Bali was perceived to have impacted Balinese attitudes towards dogs. Most of the questionnaire respondents (83 %) reported that the outbreak changed the way they kept dogs, motivating them to keep less dogs and, for some, to confine and leash them. Whilst adoption of leashing was limited to urban areas, even in rural sub-village settings, it was no longer considered socially acceptable to own more than one or two dogs; prior to rabies many dog owners had two to five dogs. Hence, through both education and fear of rabies, the outbreak was believed to have altered, to varying degrees, dog management practices with long-term implications. As one male village leader noted in a FGD:

“The fear of rabies has really changed the way we are keeping dogs [in Bali]. We now vaccinate dogs and feed them well, not like before!…Some people do not want to keep dogs anymore saying that they prefer their own safety to having a dog…and more people are starting to leash or even cage their dogs in town areas…even when Bali becomes rabies-free, people will still keep these better habits”.

### Traditional law and rabies control

Although controlling rabies is believed to be the government’s responsibility, 67 % agreed that rabies control could be optimized by instituting dog ownership regulations based on traditional law at the *banjar* level, which had already been put in place in late 2010 in some villages (according to 20 % of the questionnaire respondents). There are two types of traditional Balinese laws: *perarem* is considered to be a trial law that has an informal amount of enforcement, while *awig-awig* is a law that has been sacredly encoded through religious rituals and must be followed. The *awig-awig* laws are considered too difficult to institute for rabies control and rather the *perarem* was widely discussed and implemented in some areas. These laws included: i) accepting that your dog has to be culled if it was roaming outside the household; ii) having to pay all medical costs if someone was bitten by your dog; iii) incurring a fine if you were caught discarding unwanted puppies; and iv) paying all human funeral costs (including cremation) if your dog caused a human death. Such laws, although informal, were believed to act as *“drivers of responsibility”* in the community, motivating people to leash their dogs and vaccinate them, although the exact impact was difficult to measure in this study. In practice, dogs are free roaming and people usually recognize their dogs. The first point of the laws described above was mainly implemented in first outbreak. In later outbreaks, this point was mainly implemented when dog bite cases caused human death, in which the owners were made responsible for both human medication and traditional ceremonies (include cremation).

Socially, Bali has a unique traditional structure and social meeting groups, with traditional laws playing an important role in daily community activities. *Banjar* men, women, and youth groups meet on a regular basis, i.e. monthly or by need. These social group meetings become a space in which the community can share information on new traditional laws and their implementation. In this type of community structure, traditional leaders have the responsibility to determine whether important issues should be regulated with traditional laws to ensure community awareness and responsibility, including issues on rabies and its control.

## Discussion

The human-dog relationship in Bali is very unique and closely related with: (1) religion (Hindu), (2) culture, and (3) socioeconomic contexts. These aspects contribute to how intensive the human-dog relationship is and how important of a role dogs play in the daily lives of Balinese communities, i.e. to guard houses, be companions for people working in fields or plantations, as well their roles in religion or traditional ceremonies. These aspects also drive the practice of free-roaming dogs, which in the context of rabies can pose a high risk for spreading the disease.

Many respondents (79 %) allow their dogs to free roam and only few could handle their dogs during mass vaccination. These dog management practices need to be improved because dog vaccination can only protect the population from rabies if an adequate level of herd immunity is achieved (>70 % coverage). Moreover, the practice of discarding unwanted (and unvaccinated) female puppies will increase the number of uncontrolled free-roaming dogs (or even lead to a higher presence of stray dogs) and decrease vaccination coverage, despite vaccination campaigns reporting 70–74 % coverage [10]. Many respondents reported that their communities were keeping fewer dogs since the introduction of rabies, but such trends might change as the turnover in the dog population is believed to be really high [10]. During the FDGs, community members expressed concerns regarding the large number of puppies born every year. In the long term, these practices increase the burden of rabies control programs.

Improving dog management practices without stigmatizing community beliefs on the importance of dogs requires a better understanding of community knowledge and attitudes towards rabies and relevant control programs. Community knowledge related to rabies and the rabies control program was generally fair, but FGDs revealed that incorrect information was also circulating in the communities, i.e. rabies is curable, culling is an effective control measure for rabies, and that a fee is charged for PEP provided by the government. Hence, there is a need for more community outreach to address these information gaps and improve community knowledge.

Despite the strong influence of Hindu principles in Bali (which likely contributes to the lack of community involvement in dog culling), it appears that many people passively accepted the mass culling of dogs due to a combination of fear generated by the outbreak and wanting to abide by the government. Culling, however, was not effective in reducing the number of rabies cases [27, 28], and some vaccinated dogs were also likely culled as a result. This practice still continues at community levels in several areas because of fear and a perception generated by a previous government program. Therefore, public awareness campaigns for rabies should discourage culling.

While most questionnaire respondents understood the basics of rabies transmission, the study revealed a number of important gaps. This included: i) a lack of awareness about washing suspected rabid dog bite wounds; ii) a lack of knowledge concerning that PEP must be given prior to the development of symptoms in humans; iii) a limited knowledge that scratches could transmit rabies; and iv) a lack of emphasis on reporting rabid animals to the local livestock office to facilitate diagnostic testing and outbreak surveillance. These knowledge gaps at the local level have important public health consequences and should be included in the key messages of future campaigns. Community awareness on PEP must also be evaluated because people will want to get PEP, however, some don’t understand that the injection must be sought as soon as they are bitten (prior to symptoms) and that late treatment would be ineffective. Lack of awareness on washing dog bite wounds might be encouraged by widespread local beliefs (that existed prior to the rabies outbreak) that dog licks could heal human wounds. It might also be the reason why only 18 % of respondents answered that licks are also potential rabies transmitters. Moreover, there should be a rapid community response to dog bite cases, and this should be improved overall, not only in terms of first-aid actions and seeking medical treatment, but also ensuring reporting to the livestock office in order to prevent suspected rabid dogs from spreading the disease further. More rabies centers (reference hospitals or village/sub-district health centers) should also provide free services for PEP, as some people are still not aware that free PEP services are provided for rabies outbreak areas, such as Bali.

To improve community knowledge, better dog ownership should be encouraged, which involves educating people about leashing their dogs and regular feeding. If owners and communities can become closer with their dogs, then dogs can be handled more effectively during vaccination campaigns. In addition, it would also greatly improve the efficiency of mass vaccination programs. Since the economy poses a major challenge to the implementation of leashing and regular feeding in some rural areas, more effective strategies need to be explored.

Public awareness programs on rabies prevention and control and responsible dog ownership must be delivered regularly to ensure community knowledge improvement and to encourage behavioral changes. We found no significant relationship between gender and knowledge level or community attitudes towards rabies and its control. Based on the FGDs, men and women share equal roles and responsibilities in managing dogs. Youth and children have daily contact with dogs, for play and companionship, while adults also have contact in houses or at their workplaces. Therefore, different genders and ages have similar potential risks to contribute to the rabies spread, but could also provide valuable assistance to rabies control programs if their knowledge and attitudes are improved.

While community knowledge was generally fair and attitudes were mostly positive, the community in general still maintains free-roaming dogs. While there was a significant relationship observed between knowledge and educational level, and also between knowledge and the role in the community, there was no significant relationship observed between community knowledge, attitudes, and dog keeping practices. Dogs were generally free roaming, regardless of these variables. Therefore, campaigns should target people of all genders, ages, religions, educational backgrounds, occupations, and roles in the community, regardless of their rabies experience, to ensure appropriate information dissemination.

Once active rabies cases have been significantly reduced (as they have in Bali), elimination efforts must focus heavily on strengthening surveillance capacity and rapid response, as well as maintaining vaccination coverage [29]. Cognizant that there are both strengths and challenges involved in instituting community participation in disease control programs [30–32], it is important to consider how the Balinese community could be more actively involved in optimizing these efforts. Our study has identified a number of sociocultural aspects that can be driven to a potential community-based rabies control program, which can also go in line with a community knowledge improvement program.

First, the Balinese have a local belief that has a strong emphasis on *ahimsa* (non-violence). Such culturally embedded logics could be used to frame education efforts targeting compliance to vaccination as well as more responsible dog ownership practices. This could include more physical contact with dogs from a young age, leashing, regular feeding, and animal healthcare. Additionally, a major emphasis on education should address the practice of discarding unwanted female puppies (due to their maintenance of the stray dog population) and the reporting of rabies cases in dogs. The principle of *ahimsa* could also be used to promote a sterilization program, to explore animal welfare issues, to stop culling, to promote regular vaccination of puppies, as well as to emphasize the importance of reporting dog rabies cases to livestock officers in order to curtail further spread to dogs and humans.

Second, our research has shown unique community structures and regular social meetings (men, women, and youth) that could be engaged to facilitate increased public awareness of rabies elimination and a community’s capacity for supporting rabies control programs. Training for *banjar* or village-based volunteers (locally called *kader*), and working with *banjar* leaders through, for example, a “trainer-of-trainee” model would offer a useful pathway in raising local awareness and engaging communities in rabies control and animal health more generally. Moreover, the head of *banjar* is usually also on the board of directors for schools in the area and this means that schools can also help disseminate knowledge about rabies.

Lessons can be learnt from other similar campaigns, such as the Bohol Rabies Prevention and Elimination Project in the Philippines, which generated considerable village level involvement through thousands of volunteers, dog registration efforts, school-based education modules, and engaging legislative enforcement [33]. Bali cannot fully adopt this volunteer system because mass vaccinations in Bali have to be done by veterinarians appointed by the government. However, communities can be encouraged to participate in other ways, such as assisting vaccination programs, conducting public awareness programs, registering their dogs at the *banjar* level, and responding to any rabies outbreaks quickly. With the employment of dog-catchers, dogs were believed to have shown more “resistance” (i.e. aggressive and harder to catch) with each passing campaign and some reported to “hide” and avoid the teams as well as villagers in general. However, dog-catchers were useful for vaccinating dogs that were not owned dogs (stray dogs) or free-roaming dogs that could not be handled by their owners. Door-to-door vaccination is still relevant since not all owners can handle their dogs or bring them to vaccination points in *banjar*s due to owning a large number of dogs. At this point, volunteers can assist vaccination teams in handling the dogs or by providing baseline data of dogs registered at the *banjar* level. Formal acknowledgement and institution of community volunteers in rabies control programs needs to be considered. Volunteers may feel more appreciated and be encouraged to work harder. Moreover, volunteers will need technical assistance from the government to improve their capacity in supporting the programs to combat rabies.

Lastly, our study reveals people’s endogenous attempts to use traditional legal structures in order to increase local compliance with rabies control, which has also been reported in Tanzania [26]. With the “spirit of togetherness” (locally called *tanggung renteng*), legal codes have important social punishment dimensions and are used for a range of daily activities in Bali. Although there are certainly problems with coercive public health measures, some of which arise from community inequities [34], these are unlikely to emerge with a free state-led vaccination campaign. Such community-driven activities could be used to build support for a greater involvement, information, and guidelines for supporting elimination of rabies. Over time, it may also be useful to try and encode additional rabies-related norms into village legal structures, such as forbidding people to discard unwanted puppies, placing restrictions on the number of dogs a household can own, and making it obligatory to vaccinate dogs as many respondents stated they are willing to pay for the vaccination of puppies and accept free sterilization services, if offered by the government. However, these need to be carefully developed in collaboration with the government, NGOs, and the public to avoid misunderstandings and distortions.

As a sociocultural study that also included an analysis of risk factors of a disease, this research had number of limitations that need to be acknowledged. There was a high proportion of male respondents, determined by the convenience sampling and analysis based on village groups, and this could have biased our results, even though the study tried to complement the questionnaire data by the findings elucidated by the FGDs. Moreover, the research involved a small number of selected villages in regencies and therefore extrapolation to other areas should be made with caution. Despite the weaknesses of this research, study results and recommendations should be considered and implemented as potential pathways to optimize the rabies control program in Bali.

## Conclusion

The human-dog relationship in Bali is multifaceted. With the uniqueness of culture and local beliefs and encouraged by a socioeconomic aspect, a number of local practices were found to constitute risk factors for continued rabies spread. Community knowledge and attitudes to encourage gradual behavior changes need to be improved across different genders, age groups, educational backgrounds, religions, and roles in the community, regardless of the villages’ experiences with rabies. Community-driven activities based on sociocultural conditions and community capacity at the *banjar* and village levels were identified as potential ways for supporting the ongoing rabies control program in Bali. These include public awareness activities, vaccination, dog registration, dog population management, and rapid response to dog bites. The program also needs some recognition or acknowledgement from the governments, especially from local government as well as regular mentoring to improve and sustain community participation.

## Additional file

Additional file 1:
**Multilingual abstracts in the six official working languages of the United Nations.**
