# Supplementary material for: On dogs, people, and a rabies epidemic: results from a sociocultural study in Bali, Indonesia
Source: Infect Dis Poverty. 2015 Jun 30;4:30. doi: 10.1186/s40249-015-0061-1 (PMC4486702; doi:10.1186/s40249-015-0061-1)
Supplement: Additional file 1: — Multilingual abstracts in the six official working languages of the United Nations. [file 40249_2015_61_MOESM1_ESM.pdf]

Translation of the abstract into the six official working languages of the United Nations

عن الكلاب، الناس، وفاشية داء الكَلَب: نتائج دراسة اجتماعية ثقافية في بالي، إندونيسيا

م . د. و. ودياستوتي، ك.ل.باردوش، أس. سُنندار، سي باسريل، إي. باسونو، إيه. جاتيكوسوماه، ر. إيه. أريف، إيه. إيه. جي. بوترا، إيه. روكمنتارا، إيه. تي. إس. أستوبيانجستي، آي. وليانتو، آي كي. جي. نتاكيسوما، آي. بي. سومنترا، دي. جريس، إف. أنجر، جي. جلبرت

الموجـــز

الخلفية: بعد أن كانت خالية من داء الكلب، شهدت بالي فاشية من هذا المرض عام 2008، تسببت منذ ذلك الوقت بحدوث عدد كبير من الوفيات البشرية. ردا على ذلك، نفذت حملة واسعة لاعدام الكلاب وتطعيمها. ولتقييم التدخلات المجتمعية الممكنة لتحسين مكافحة داء الكلب، أجرينا دراسة لاستكشاف العلاقة بين الكلاب، داء الكلب، والمجتمع في مدينة بالي. تهدف هذه الدراسة إلى: أ) فهم العلاقة بين الإنسان والكلب في بالي. ب) استكشاف المعرفة، والمواقف، والممارسات المحلية المتعلقة بداء الكلب. وج) تقييم الأنشطة المجتمعية الممكنة لتحسين مكافحة داء الكلب ومراقبته.

الطرق: الدراسة التي أجريت بين شهري فبراير (شباط) ويونيو (حزيران) 2011، جمعت بين استبيان (عدد المشاركين = 300؛ فاصلة الثقة =95%؛ هامش الخطأ = 5%) ونقاشات جماعية مركزة في 10 قرى تقع في مقاطعات دنباسار، يانيار، وكرنغاسم. تضمن الاستبيان مقياس ليكرت لتقييم معرفة المجتمع وسلوكياته. منح الجواب الصحيح أو الإيجابي 3 نقاط، في حين منحت الإجابات السلبية/الخاطئة أو غير المؤكدة 0. تم تصنيف معرفة المستجيبين إلى جيدة (معدل > 40 نقطة)، متوسطة (معدل 20 – 40 نقطة)، أو سيئة (معدل < 20 نقطة)، استنادا إلى معدل درجات أقصى قدره 60 نقطة. وتم تصنيف سلوك المستجيبين إلى إيجابي (معدل > 26 نقطة)، حيادي (معدل 13 – 26 نقطة)، أو سلبي (معدل < 13 نقطة)، استنادا إلى معدل درجات أقصى قدره 39 نقطة. عقدت نقاشات جماعية مركزة تضم الجنسين في كل قرية فرعية، وشملت كل منها 7 – 15 مشاركا لاستكمال نتائج الاستبيان. وفي رحلة متابعة بحثية في منتصف 2013، تم تثليث تحليل البيانات والتحقق من صحتها باستخدام لقاءات شبه منظمة. أجري تحليل وصفي لبيانات الاستبيان باستخدام SPSS 17.0، في حين أجري تحليل يدوي للبنانات النوعية من اللقاءات والنقاشات الجماعية المركزة حسب الطرق المقبولة لترميز وكتابة الملاحظات. ثم استعمل اختبار خي مربع لتحليل العلاقات الإحصائية بين معرفة المستجيب وسلوكياته.

النتائج: من بين 300 مستجيب، كانت غالبيتهم من الذكور (82%)، الهندوس (99%)، المتزوجين (96%)، ويتجاوز عمرهم 30 سنة (92%)، ويمتلكون كلابا (72%). كان الدافع لاقتناء الكلب ثقافي، ويتعلق بالذوق الشخصي، والمهنة، وكانت الكلاب تستخدم للحراسة (85%)، أو كحيوانات مصاحبة (27%)، وفي بعض الأحيان لواجبات دينية أو تقليدية. ربطا بمعتقداتهم الثقافية والمحلية، ومع تحولها بالنتيجة إلى أسلوب حياة، احتفظ 79% من المستجيبين بكلاب طليقة. ومع ظهور فاشية داء الكَلب في بالي وازدياد شعبية السلالات الغربية، أصبح اقتناء الكلاب بطريقة تنطوي على مسؤولة أكبر (التقييد وتحديد الحركة والتغذية المنتظمة) أكثر تقبلا وغيّر مفاهيم المجتمع حيال اقتناء الكلاب، على الرغم من تعذّر قياس استدامة هذه الممارسة. بالإضافة إلى ذلك، شكل الوضع الاقتصادي مشاكل كبرى في المناطق الريفية. كان مستوى معرفة المجتمع بداء الكَلب وما يرافقه من برامج السيطرة عليه متواضعا بشكل عام وكانت سلوكيات المجتمع إيجابية. ومع ذلك، ما تزال المعرفة، والمواقف، والممارسات المجتمعية المحلية بحاجة إلى تحسين. ذكر ما مجموعه 74% من المستجيبين أنهم قاموا بتطعيم كلابهم عام 2011، ولكن وجد أن عددا قليلا فقط (12%) أبلغ عن إصابة الحيوانات المسعورة لضباط المواشي واعتقد عدد كبير (62%) أن غسل جرح العضة لم يكن ذا أهمية. وعلاوة على ذلك، تستمر ممارسات اقتناء الكلاب الطليقة والتخلص من الجراء الإناث غير المرغوب فيها، وربما تخلق صعوبات للقضاء على داء الكلب لأن هذه الممارسات من المحتمل أن تزيد من عدد الكلاب الضالة. لقد حددنا ثلاثة جوانب اجتماعية وثقافية كبيرة يمكنها التدخل لدوافع اجتماعية لتحسين الجهود المبذولة حاليا للقضاء على داء الكلب: دمج المفاهيم المحلية لـ *ahimsa* (اللاعنف) في حملات التوعية، وإشراك المجتمعات المحلية من خلال النظام الاجتماعي السياسي للقرى والنواحي المحلية ، والعمل مع البنى القانونية التقليدية لزيادة الامتثال المحلي بمكافحة داء الكلب.

الخاتمة: هناك عدة وجوه للعلاقة بين البشر والكلاب في بالي. ونظرا لفرادة التقاليد والمعتقدات المحلية، وبتشجيع من الناحية الاجتماعية الاقتصادية، وجد أن عدة ممارسات محلية تشكل عوامل خطورة لاستمرار انتشار داء الكَلب. المعرفة والمواقف المجتمعية، التي يمكنها أن تؤدي بالنتيجة إلى تغيّرات سلوكية، بحاجة لتحسين عند الجنسين ولدى كافة الأعمار والخلفيات الثقافية والأدوار الاجتماعية بصرف النظر عن تجربة القرية التي ينتمي إليها المشارك مع داء الكَلب. وعلاوة على ذلك، فالنشاطات ذات الدوافع المجتمعية والمستندة إلى التكيف الاقتصادي الاجتماعي وقدرة المجتمع على التقبل على مستوى القرى والنواحي المحلية، مثل نشاطات التوعية العامة، تطعيم وتسجيل الكلاب، وادارة أعداد الكلاب، والاستجابة السريعة حيال عضات الكلاب، اعتبرت جميعا قادرة على تكمّلة برامج السيطرة على داء الكَلب في بالي. البرنامج أيضا بحاجة لاعتراف من الحكومات، والمحاية منها بوجه خاص إضافة إلى المراقبة المستمرة لتحسين وإدامة المشاركة المجتمعية.

Translated from English version into Arabic by Lina SM, through

TRANSLATORS WITHOUT BORDERS

从社会文化学的角度研究印度尼西亚巴厘岛狂犬病疫情与人、犬的关系

M.D.W. Widyastuti, K.L. Bardosh, S. Sunandar, C. Basri1, E. Basuno, A. Jatikusumah, R.A. Arief, A.A.G. Putra, A. Rukmantara, A.T.S. Estoepangestie, I. Willyanto, I.K.G. Natakesuma, I.P. Sumantra, D. Grace, F. Unger, J. Gilbert

摘要

引言：2008 年，巴厘岛爆发了一场空前的狂犬病疫情，导致大批民众死亡。为此，相关部门对当地犬只进行了大规模捕杀和疫苗接种。为评估潜在社区驱动干预措施对控制狂犬病疫情的促进作用，我们研究了犬、狂犬病与巴里社区居民之间的关系。本研究旨在：ｉ）了解巴厘岛人犬关系；ii）了解当地居民的狂犬病知识、态度和行为；iii）评估潜在社区驱动措施以优化狂犬病控制和监测工作。

方法：2011 年 2 月至 5 月，在登巴萨、吉安雅和卡朗加沙摄政区的 10 个村庄里开展问卷调查（n=300; CI=95%;；误差=5%）和专题小组讨论。本研究运用问卷中的利开特式量表评估社区知识和态度，正确或积极答案得 3 分，消极、错误和不确定答案均为零分。狂犬病知识总分为 60 分，分为 3 类：好（>40 分）、中（20~40 分）、差（<20）。态度总分为 39 分，也分为 3 类：积极（>26 分）、中立（13~26 分）、消极（<13 分）。每个村组的男女混合专题小组讨论（5~7 人）补充调查问卷结果。2013 年中期，我们进行了随访调查，所得数据进行三角化处理分析，并运用半结构化访谈验证。采用 SPSS 17.0 统计软件分析调查问卷结果。使用公认的编码和备忘录记录人工分析访谈和专题小组讨论所得定性资料。采用卡方检验分析受访者知识和态度之间的统计学关系。

结果：300 名受访者主要为男性(82%)、养犬者(72%)、印度教教徒(99%)、已婚人士(96%)、30 岁以上(92%)。养狗的动机有文化影响、个人爱好和犬的作用（守卫：85%和陪护：27%），也有人因宗教或传统责任养犬(2%)。79%受访者的遛狗行为始于当地文化信仰，后逐渐成为个人生活习惯。狂犬病的爆发和西方犬种的流行促使巴厘岛人采取更负责的养犬方式（牵着、圈养、定期饲养）、转变社区养狗观念，尽管目前难以明确这种养犬方式的持久度。此外，边远地区的经济状况也是一大难题。当地居民对狂犬病疫情及相关措施有所了解，态度积极。但是，社区居民的狂犬病知识、态度和行为仍需提高。据报道，2011 年 74%的受访者为犬只注射过狂犬疫苗，但仅少数人上报患狂犬病的动物(12%)，而多数人认为并无必要清洗咬伤伤口(62%)。而且，由于一些人随意遛狗和丢弃母狗造成走失犬只增多，这阻碍了狂犬病消除行动。我们从社会文化三个方面对目前社区驱动措施进行了评估，即将当地非暴力概念整合至教育活动，通过当地村级社会政治系统参与社区活动，与传统执法部门联合加强当地的狂犬病控制。

结论：巴厘岛人犬关系错综复杂。由于当地信仰和文化的独特性，加之受社会经济的影响，当地人的一些行为仍然可能导致狂犬病的传播。不管个人是否经历过狂犬病，社区总体认知和态度总会转变个人行为，为此，仍需从性别、年龄、教育背景、社区角色方面提高人们对狂犬病的了解程度，端正个人态度。进一步讲，基于社会文化条件和社区能力的村组级社区驱动措施将会大力促进巴厘岛狂犬病防控工作，如公共教育活动、接种疫苗、犬只登记与数量管理、及时清理犬只咬伤伤口等。本项目也需得到政府部门的大力支持和认可，特别当地政府的定期指导和当地社区的持续参与。

Translated from English version into Chinese by Chen Jin, edited by Yang Pin, through

CHINESE CENTER FOR DISEASE CONTROL AND PREVENTION
NATIONAL INSTITUTE OF PARASITIC DISEASES

Résultats d'une étude socio-culturelle menée à Bali, en Indonésie, portant sur la population locale, les chiens et l’épidémie de rage

M.D.W. Widyastuti, K.L. Bardosh, S. Sunandar, C. Basri1, E. Basuno, A. Jatikusumah, R.A. Arief, A.A.G. Putra, A. Rukmantara, A.T.S. Estoepangestie, I. Willyanto, I.K.G. Natakesuma, I.P. Sumantra, D. Grace, F. Unger, J. Gilbert

EXTRAIT

Contexte : Alors que Bali n'était jusque là pas affectée par la rage, une épidémie a démarré sur l'île en 2008, qui a causé de nombreux décès humains depuis. Il en a résulté une extermination massive de chiens ainsi que des campagnes de vaccinations. Afin d'évaluer les interventions communautaires possibles dans le but d'optimiser la lutte contre la rage, nous avons mené une étude sur les relations entre les chiens, la rage et la communauté balinaise. Les objectifs de cette étude étaient de :i) comprendre les relations homme-chien à Bali ;ii) étudier les connaissances, attitudes et pratiques (CAP) locales liées à la rage ; et iii) évaluer le potentiel pour des actions communautaires d'optimisation de la surveillance et de la lutte contre la rage.

Méthodologie : Menée entre février et juin 2011, l'étude a associé un questionnaire (n=300; intervalle de confiance=95 %; marge d'erreur=5 %) et des groupes de discussion dans 10 villages des régions de Denpasar, Gianyar, et Karangasem. Le questionnaire comprenait une échelle de Likert pour évaluer les connaissances et attitudes de la communauté. Une réponse positive correcte valait trois points, tandis que les réponses négatives/fausses ou incertaines valaient zéro point. Sur un maximum de 60 points, les connaissances des personnes interrogées étaient classées selon trois catégories : bonnes (score > 40), moyennes (score entre 20 et 40) ou faibles (score < 20). Sur un maximum de 39 points, les attitudes des personnes interrogées étaient classées selon trois catégories : positives (score > 26), neutre (score entre 13 et 26) ou négatives (score <13). Pour compléter les résultats des questionnaires, des groupes de discussion mixtes, comprenant de 7 à 15 participants, ont été menés dans chacun des hameaux (*banjar*). Lors d'un voyage de recherche de suivi mi-2013, l'analyse des données a été triangulée et validée par le biais d'entretiens semi-directifs. Les données des questionnaire ont été analysées à l'aide du logiciel SPSS 17.0, tandis que les données qualitatives issues des entretiens et des groupes de discussion ont été analysées manuellement suivant des méthodes reconnues de codage et d'écriture de mémos. Le test du χ^2 a ensuite été utilisé pour analyser la corrélation statistique entre les connaissances et les attitudes des personnes interrogées.

Résultats : Sur un total de 300 personnes interrogées, la majorité des personnes étaient des hommes (82 %), hindoues (99 %), mariées (96 %), âgées de plus de 30 ans (92 %) et propriétaires de chiens (72 %). Les propriétaires de chiens le deviennent en raison de leur culture, de leurs goûts personnels, et de leur travail. Les chiens peuvent être des chiens de garde (85 %) et des animaux de compagnie (27 %). Posséder un chien est parfois lié à des obligations religieuses ou traditionnelles (2 %). Du fait de leur culture et des croyances locales, 79 % des interrogés gardaient leurs chiens en liberté, ce qui est par la suite devenu coutumier. Suite à l'épidémie survenue à Bali, et à la montée en popularité des chiens de races occidentales, la responsabilisation des propriétaires de chiens (tenir les chiens en laisse, les garder enfermés, les nourrir de manière régulière) est devenue plus acceptable et a changé les perceptions communautaires sur la façon de s'occuper des chiens même si la durabilité de telles pratiques ne peut pas être évaluée. La situation économique a aussi posé des problèmes supplémentaires dans les zones rurales. Les connaissances communautaires sur la rage et les programmes de lutte contre la rage étaient généralement de niveau moyen et les attitudes communautaires positives. Cependant les CAP communautaires doivent encore être améliorées. Au total, 74 % des personnes interrogées ont rapporté avoir fait vacciner leurs chiens en 2011, mais seule une minorité a rapporté aux autorités les animaux atteints de la rage (12 %) et une proportion importante était convaincue qu'il n'est pas important de laver une morsure (62 %). De plus, les pratiques constituant à garder les chiens en liberté et à se débarrasser des chiots femelles non voulues perdurent et pourraient créer des difficultés pour l'élimination de la rage, étant données que ces pratiques contribuent à augmenter le nombre de chiens errants. Nous avons identifié trois aspects socioculturels majeurs ayant le potentiel d'être intégrés dans des interventions communautaires ayant pour but d'optimiser les efforts actuels de lutte contre la rage : intégrer les notions locales d'*ahimsa* (non-violence) dans des compagnes éducatives, engager les communautés par le biais du système sociopolitique local de *banjar* et travailler avec les structures juridiques traditionnelles pour augmenter le respect local des programmes de lutte contre la rage.

Conclusion : Les relations homme-chien ont de nombreux visages à Bali. En raison d'une culture unique et de croyances locales, de plus renforcées par un aspect socio-économique, de nombreuses pratiques locales sont considérées comme des facteurs de risque de prolongation de l'épidémie de rage. Les connaissances et attitudes communautaires, qui peuvent donner lieu à des changements de

comportement, doivent être améliorés pour les différents sexes et une variété d'âges, de niveaux de scolarité et de rôles dans la communauté, quelle que soit l'expérience du village donné avec la rage. En outre, nous avons identifié que les activités communautaires liées à des conditionnements socio-culturels et que les capacités communautaires au niveau des *banjar* et des villages pour des actions telles que la sensibilisation des populations, les vaccinations, la déclaration des chiens, la gestion de la population canine et une réponse rapide en cas de morsure devraient pouvoir compléter le programme de lutte contre la rage à Bali. Le programme doit aussi être soutenu et reconnu le gouvernement, en particulier par les autorités locales et parrainé régulièrement pour maintenir et augmenter la participation des communautés.

Translated from English version into French by Fabienne Perrin, through

TRANSLATORS
WITHOUT BORDERS

О собаках, людях и эпидемии бешенства: Результаты социокультурного исследования в Бали, Индонезия

М.Д.У. Уидьястути, К.Л. Бардош, С. Сунандар, К. Бастрил, Е. Базуно, А. Ятикусума, Р.А. Ариеф, А.А.Г. Путра, А. Рукмантара, А.Т.С. Эстоепангестие, И. Уильянто, И.К.Г. Натакесума, И.П. Сумантра, Д. Грейс, Ф. Унгер, Дж. Гилберт

РЕФЕРАТ

История вопроса: Хотя в прошлом в Бали не наблюдалось случаев бешенства, в 2008 году произошла вспышка эпидемии, вызвавшая с тех пор большое количество человеческих жертв. В ответ был произведен как массовый селекционный отлов собак, так и вакцинация. Для оценки потенциальных мероприятий, осуществляемых местными общинами с целью оптимизации контроля распространения бешенства, были исследованы взаимосвязи между собаками, бешенством и населением Бали. Целями данного исследования являлось: i) понять характер взаимоотношений между собаками и людьми в Бали; ii) изучить местные осведомлённость, отношение и практику в отношении бешенства; и iii) осуществить оценку потенциальных мероприятий, осуществляемых местными общинами, с целью оптимизации контроля распространения и надзора за бешенством.

Методы: Исследование проводилось в феврале-июне 2011 года и включало анкетирование (n=300; CI =95% (доверительный интервал при доверительной вероятности 0,95); предел погрешности=5%) и целенаправленные групповые обсуждения в 10 деревнях в округах Денпасар, Джианьяр и Карангасем. Анкета включала шкалу Лайкерта для оценки осведомленности и установок населения. За правильный или положительный ответ присваивалось три балла, а отрицательные/неправильные и неясные ответы получали ноль баллов. Осведомленность опрашиваемых оценивалась как хорошая (сумма баллов >40), удовлетворительная (сумма баллов 20–40) или неудовлетворительная (сумма баллов <20) при максимальной общей сумме баллов 60. Установка опрашиваемых классифицировалась как положительная (сумма баллов >26), нейтральная (сумма баллов 13–26) или отрицательная (сумма баллов <13) при максимальной общей сумме баллов 39. Для дополнения результатов анкетирования в каждом поселении (*banjar*) проводились целенаправленные групповые обсуждения для лиц обоего пола, в каждом из которых участвовало 7–15 человек. Во время контрольного исследования в середине 2013 года анализ полученных данных был пересмотрен под разными углами и обоснован с помощью полуструктурированных интервью. Данные анкетирования были подвергнуты описательному анализу с помощью пакета программ обработки статистических данных общественных наук SPSS 17.0, а количественные данные, полученные в ходе интервью и целенаправленных групповых обсуждений, анализировались вручную в соответствии с принятыми методами кодирования и составления

письменных справок. Для анализа статистической зависимости между осведомленностью и установками опрашиваемых использовался метод распределения хи-квадрат.

Результаты: Из 300 опрашиваемых преобладающее большинство было мужского пола (82%), индуисты (99%), состояло в браке (96%), в возрасте старше 30 лет (92%) и имело собак (72%). Владение собаками обуславливалось культурными традициями, личной предрасположенностью и практическими целями, поскольку собаки использовались для охраны (85%) и как домашние животные (27%), и в некоторых случаях было связано с религиозным долгом или основанными на традициях обязательствами (2%). В соответствии с местной культурой и поверьями, ставшими образом жизни, 79% опрашиваемых держали полубродячих собак. Вспышка эпидемии бешенства в Бали и распространение западных пород собак привело к тому, что стало более приемлемым более ответственное отношение к владению собаками (привязывание, ограничение движения и регулярное кормление) и изменились представления населения о содержании собак, хотя и невозможно оценить долговечность и устойчивость новых обычаев. Кроме того в связи с экономическим положением в сельских областях наблюдаются серьезные проблемы. Уровень осведомленности населения о бешенстве и о программах контроля бешенства в основном достаточный, и установки населения были положительными. Однако, осведомлённость, отношение и практика населения нуждаются в улучшении. Всего 74% опрашиваемых сообщили, что они сделали прививки своим собакам в 2011 году, но было обнаружено, что мало кто сообщает о бешеных животных зоотехническим инспекторам (12%), а значительное количество опрошенных считало, что совершенно необязательно промывать укусы (62%). Кроме того, продолжающаяся практика содержания бродячих собак и избавления от нежеланных щенков женского пола по всей вероятности препятствует устранению бешенства, поскольку это ведет к потенциальному росту популяции бродячих собак. Нами определены три основных социально-культурных аспекта, обладающих потенциалом для мероприятий, осуществляемых местными общинами с целью оптимизации усилий по устранению бешенства: использование в просветительных кампаниях местного понятия *ahimsa* (ненасилие), вовлечение местных сообществ посредством местной социально-политической системы *banjar* (поселение) и работа с традиционными правовыми структурами для повышения действенности мероприятий по контролю бешенства.

Выводы: Характер взаимоотношений между собаками и людьми в Бали имеет многогранный характер. Было установлено, что часть местных обычаев, основанных на уникальности культуры и местных поверий и поддерживаемых социально-экономическими факторами, способствует продолжающемуся распространению бешенства. Необходимо повысить осведомленность и изменить отношение населения, что должно привести к изменениям поведения, причем это должно быть сделано в разных гендерных и возрастных группах, у лиц с разным образовательным уровнем и ролями в сообществе и независимо от конкретного опыта каждой отдельной деревни по отношению к бешенству. Кроме того, было установлено, что программа по контролю бешенства в Бали может быть успешно дополнена общественными видами деятельности, основанными на социально-культурном обусловливании и активности общин на уровне поселений (*Banjar)* и деревень, как, например, просветительская деятельность, вакцинация, регистрация собак, контроль их популяций и экстренная помощь при укусах. Программа также нуждается в признании и поддержке со стороны правительства, особенно местного, а также в регулярном менторстве для повышения и поддержки участия в ней общественности.

Translated from English version into Russian by Alena Hrybouskaya, through

TRANSLATORS
WITHOUT BORDERS

Sobre perros, gente y la epidemia de rabia: Resultados de un estudio sociocultural en Bali, Indonesia

M.D.W. Widyastuti, K.L. Bardosh, S. Sunandar, C. Basri1, E. Basuno, A. Jatikusumah, R.A. Arief, A.A.G. Putra, A. Rukmantara, A.T.S. Estoepangestie, I. Willyanto, I.K.G. Natakesuma, I.P. Sumantra, D. Grace, F. Unger, J. Gilbert

RESUMEN

Antecedentes: Previamente libre de rabia, Bali experimentó un brote en el año 2008, el cual desde entonces ha producido una gran cantidad de muertes humanas. En respuesta, se implementaron campañas masivas de sacrificio de perros y vacunación. Para poder evaluar posibles intervenciones promovidas por la comunidad para la optimización del control de la rabia, hemos llevado a cabo un estudio que explora la relación entre perros, rabia y la comunidad de Bali. Los objetivos del presente estudio fueron: i) entender la relación ser humano-perro en Bali; ii) explorar el conocimiento, actitudes y prácticas (KAPs) locales en cuanto a la rabia; y iii) evaluar posibles actividades promovidas por la comunidad para optimizar el control y monitoreo de la rabia.

Métodos: El estudio se llevó a cabo entre febrero y junio de 2011. Combinó un cuestionario (n=300 IC=95%; margen de error=5%) y grupos de discusión enfocados (FGDs) en 10 aldeas en las prefecturas de Denpasar, Gianyar y Karangasem. El cuestionario incluía una escala Likert para evaluar el conocimiento y actitudes de la comunidad. Se asignaban tres puntos a una respuesta correcta o positiva, mientras que las respuestas negativas/incorrectas y las respuestas inciertas recibían cero punto. El conocimiento de los encuestados se categorizó como bueno (puntaje >40), regular (puntaje 20-40), o pobre (puntaje <20), basado en un puntaje total máximo de 60. Las actitudes de los encuestados se categorizaron como positivas (puntaje >26), neutras (puntaje 13-26), o negativas (puntaje <13), basado en un puntaje total máximo de 39. Para complementar los resultados del cuestionario se llevaron a cabo grupos de discusión enfocados mixtos, de ambos géneros en cada sub-aldea(banjar), cada uno con 7 a 15 participantes. En un viaje de investigación de seguimiento a mediados del 2013, se trianguló y validó el análisis de la información mediante el uso de entrevistas semi-estructuradas. La información de los cuestionarios fue analizada descriptivamente con SPSS 17.0, y la información cualitativa de las entrevistas y de los grupos de discusión enfocados fueron analizados manualmente de acuerdo a métodos aceptados de codificación y escritura de memo. Luego se utilizó la prueba de chi-cuadrado para analizar las relaciones estadísticas entre conocimiento y actitudes de los encuestados.

Resultados: De los 300 encuestados, la mayoría eran hombres (82%), hindúes (99%), casados (96%), de más de 30 años de edad (92%), y tenían perros (72%). La tenencia de perros estaba motivada por la cultura, gusto personal, y la función, con los perros siendo utilizados como guardias (85%) y animales de compañía (27%), y a veces estaba relacionado con obligaciones religiosas o tradicionales (2%). Con relación a su cultura y creencias locales, y eventualmente a pasar a ser su forma de vida, 79% de los encuestados tenían a sus perros libres. Con el brote de rabia en Bali y las razas occidentales cada vez más populares, la tenencia responsable de perros (uso de correa, encierro, alimentación regular) ha pasado a ser más aceptable y ha cambiado la percepción de la comunidad sobre la tenencia de perros, incluso cuando no puede medirse la sostenibilidad de dicha práctica. Asimismo, la situación económica representaba mayores problemas en las zonas rurales. Los niveles de conocimiento de la comunidad sobre la rabia y sus programas de control asociados por lo general eran favorables y las actitudes de la comunidad positivas. Sin embargo, todavía necesitan mejorarse el conocimiento, actitudes y prácticas. Un total de 74% encuestados reportaron haber vacunado a sus perros en el 2011, pero solo pocos reportaban los animales rabiosos a los oficiales del ganado (12%) y una cantidad significativa pensaba que no era importante lavar una herida por mordida (62%). Es más, las prácticas de los perros merodeando libremente, y el descarte de cachorros hembras todavía continuaba y posiblemente crea dificultades para la eliminación de la rabia ya que estas prácticas potencialmente aumentan la población de perros callejeros. Identificamos tres aspectos socioculturales importantes con potencial para intervenciones promovidas por la comunidad para optimizar los esfuerzos actuales de eliminación de la rabia: integración de las nociones locales de *ahimsa* (no-violencia) en las campañas educativas, integración a las comunidades a través del sistema sociopolítico local *banjar*, y el trabajo con las estructuras legales tradicionales para incrementar el cumplimiento local con el control de la rabia.

Conclusión: La relación ser humano-perro en Bali es multifacética. Debido a la singularidad de la cultura y las creencias locales, y estimulado por un aspecto socioeconómico, se encontró que una cierta cantidad de prácticas locales constituían factores de riesgo para una transmisión continua de la rabia. El conocimiento y las actitudes de la comunidad, que pueden constantemente resultar en cambios de comportamiento, necesitan mejorarse en todos los géneros, edades, niveles educativos, y roles en la comunidad, sin importar la experiencia de cada aldea individual con la rabia. Es más, las actividades promovidas por la comunidad basadas en el condicionamiento sociocultural y la capacidad de la comunidad en el *banjar* y los niveles de las aldeas, como las actividades de concientización pública, vacunación, registro de perros, manejo de la población de perros, y la respuesta rápida ante las mordidas de perro, fueron identificadas como capaces de complementar el programa de control para la rabia en Bali. El programa también necesita reconocimiento y aceptación por parte de los gobiernos, en particular el gobierno local así como programas de orientación periódicos para mantener la participación de la comunidad.

Translated from English version into Spanish by Maria Alejandra Aguada, through

TRANSLATORS
WITHOUT BORDERS
